# *Trichoderma longibrachiatum*-Associated Skin Inflammation and Atypical Hyperplasia in Mouse

**DOI:** 10.3389/fmed.2022.865722

**Published:** 2022-04-28

**Authors:** Gongjie Zhang, Dongming Li

**Affiliations:** Division of Dermatology and Mycological Lab, Peking University Third Hospital, Beijing, China

**Keywords:** *Trichoderma longibrachiatum*, trichodermasis, proliferation, mouse experiment, carcinoma

## Abstract

**Background:**

The relationship between infection and tumors has attracted increasing attention. *Trichoderma* spp. are often isolated from tumors. However, their potential role remains unclear. We recently reported the isolation of *Trichoderma longibrachiatum* from a patient with pulmonary spindle cell carcinoma that was confirmed as primary infection by application of laser capture microdissection and polymerase chain reaction. To explore whether the strain is pathogenic and whether it can cause atypical cell proliferation and infiltration of NK cells and T cells, we designed a mouse infection experiment.

**Methods:**

Twelve ICR mice were randomly separated into three groups. Cyclophosphamide was used to inhibit the immunity of mice. A mouse model of Trichoderma infection was successfully established by intracutaneous injection on the back skin with a suspension of strain PKUT180420015. The pathological manifestations of Trichoderma infection and the interaction between immune cells and fungi were observed by histopathology, immunohistochemistry and intensive fungal staining. Reisolation of the fungus was observed by infected tissue culture. The inoculated sites exhibited swelling 3 days after inoculation, and ulcers developed at approximately 14 days. Skin specimens were obtained and then cultured at 3, 7, and 14 days after inoculation. We selected mice 14 days after inoculation in Group 3, whose ulcers were the most typical, for histological analysis.

**Results:**

Inflammation, angioinvasion and necrosis were observed. Immunohistochemistry showed positive markers of Ki67, CD3, CD56, GZMB, and PRF. Periodic acid-Schiff staining, periodic acid-silver methenamine staining, and Calcofluor staining showed fungal spores in the vascular lumen, vascular walls and around the blood vessels.

**Conclusions:**

Our studies showed that a *T. longibrachiatum* strain (PKUT180420015) isolated from a biopsy specimen in a patient with pulmonary spindle cell carcinoma could induce atypical hyperplasia, with the expression of Ki67, CD3, CD56, GZMB, and PRF in mice. These data indicate that the fungus may be involved in inducing atypical hyperplasia or tumorigenesis.

## Introduction

Clinical manifestations of trichodermasis include pneumonia (1), rhino-orbital-cerebral mycosis, oral infection, otitis externa, sinusitis, brain abscess, stomatitis, mediastinitis and peritonitis (2), endocarditis (3), skin and skin structural infection, keratitis, and septic shock (4). *Trichoderma* spp. are often isolated from tumors. Much has been learned about the relationship between fungi and cancer. Among them, *Aspergillus flavus* is common, and its metabolite aflatoxin may lead to liver cancer. *Trichoderma* is similar to *Aspergillus* in morphology and evolution. There have been no published reports that *Trichoderma* or its toxin can induce carcinoma. We recently reported the isolation of *Trichoderma longibrachiatum* from the tissue of a patient with pulmonary spindle cell carcinoma that was confirmed as primary infection by laser capture microdissection and polymerase chain reaction (5). However, its potential role is unclear. To explore whether the strain can cause atypical cell proliferation, we designed a preliminary mouse infection experiment.

## Materials and Methods

### Isolates

The green filamentous fungus PKUT180420015 was isolated from the tissue of a patient with pulmonary spindle cell carcinoma at Peking University Third Hospital. The isolate was grown on Sabouraud's dextrose agar (SDA) at 28°C for 7 days. Conidia were harvested by gently washing the surface of the slants with saline. The suspension was filtered through a sterile 40 μm cell strainer, and the number of conidia was adjusted to 5 × 10^7^ colony-forming units per milliliter (CFU/ml) by counting the spores in a hemocytometer and subsequently verifying these results through quantitative colony counts on PDA plates. Resting conidia were immediately used or stored at +4°C (6).

### Mice

Twelve ICR mice (male, 18–22 g, 6–8 weeks) were purchased from Beijing Keyu Animal Breeding Center Company (Beijing). All mice were bred in the Animal Experimental Department, Fourth Medical Center, PLA General Hospital under specific-pathogen-free (SPF) conditions. The 12 mice were randomly divided into four groups, with three mice in each group.

### Establishment of an Immunosuppression Model

Mice in Group 1, Group 2, and Group 3 were immunosuppressed by intraperitoneal injection of cyclophosphamide (80, 90, 100 mg/kg·d, respectively) for 3 days. Mice in Group 4, the control group, received an intraperitoneal injection of cyclophosphamide at 100 mg/kg·d (6).

### Establishment of the Skin Infection Model

Mice in Groups 1, 2, and 3 between 6 and 8 weeks of age were infected by intracutaneous injection on the skin of the back with a suspension of *T. longibrachiatum* (0.02 ml). Group 4 was intracutaneously injected with saline (0.02 ml) as the normal control. To evaluate the effect of this study on the mental state of mice, we observed their appetite, water consumption and cage activity.

### Access and Processing of Skin Specimens

At 3, 7, and 14 days after inoculation, one mouse was euthanized in each group by the atlantoaxial dislocation method. Skin specimens from the inoculation site were obtained for fungal culture in SDA at 28°C for 7 days The skin specimens of the mice 14 days after inoculation in Group 3, whose ulcer was the most typical, were embedded in paraffin and serially cut for histopathological examination (hematoxylin-eosin staining, periodic acid-Schiff staining, and periodic acid-silver methenamine staining).

### Antibodies

The primary antibodies used for immunohistochemical staining included the following: anti-CD56/NCAM (neural cell adhesion molecule), anti-Ki67 and anti-granzyme B (all from Abcam, Boston, USA), anti-perforin and anti-CD3ε (all from Cell Signaling Technology, Danvers, USA). Anti-rabbit (Zhongshan Gold Bridge, Beijing, China) was used as the secondary antibody for immunohistochemical staining. Unstained cells were used as negative controls, and mouse spleen cells were used as positive controls.

### Immunohistochemical Staining

The paraffin sections were dried at 60°C for 4 h, and paraffin was removed by immersing them into dimethylbenzene substitutes (3 × 15 min), absolute ethanol (2 × 5 min), 95% ethanol (2 × 5 min), and 80% ethanol (5 min) and then distilled water for 2 min. Then, the samples were incubated with 3% peroxide-methanol in a light-resistant container at room temperature for 10 min for endogenous peroxidase ablation. All following steps were carried out in a moist chamber: The samples were rinsed 3 times with clean water, 3 times with distilled water and 3 times in PBS (phosphate buffer solution, pH 7.4). Antigens were retrieved by high pressure and high temperature antigen retrieval *via* immersion in citric acid buffer (pH 6.0). The slides were rinsed in PBS for 3 × 5 min. The anti-CD56/NCAM, anti-Ki67, anti-CD3ε, anti-perforin and anti-granzyme B antibodies were diluted to 1:2,000, 1:600, 1:150, 1:600, and 1:1,200, respectively. They were dropped (50–100 μl) onto slides and incubated at 37°C for 2 h. Then, 50–100 μl of secondary antibody solution was dropped onto the slides and incubated for 30 min at room temperature. The slides were rinsed in PBS for another 3 × 5 min. All slides were incubated with 3,3-diaminobenzidine (DAB, Zhongshan Gold Bridge, Beijing, China) for coloration. The cell nuclei were counterstained with hematoxylin. Then, the slides were immersed in 95% ethanol (2 × 2 min), absolute ethanol (2 × 2 min), and dimethylbenzene substitutes (2 × 5 min). Finally, the slides were dehydrated and integrated with neutral balsam.

## Results

### Skin Lesions

**Figure 1:**
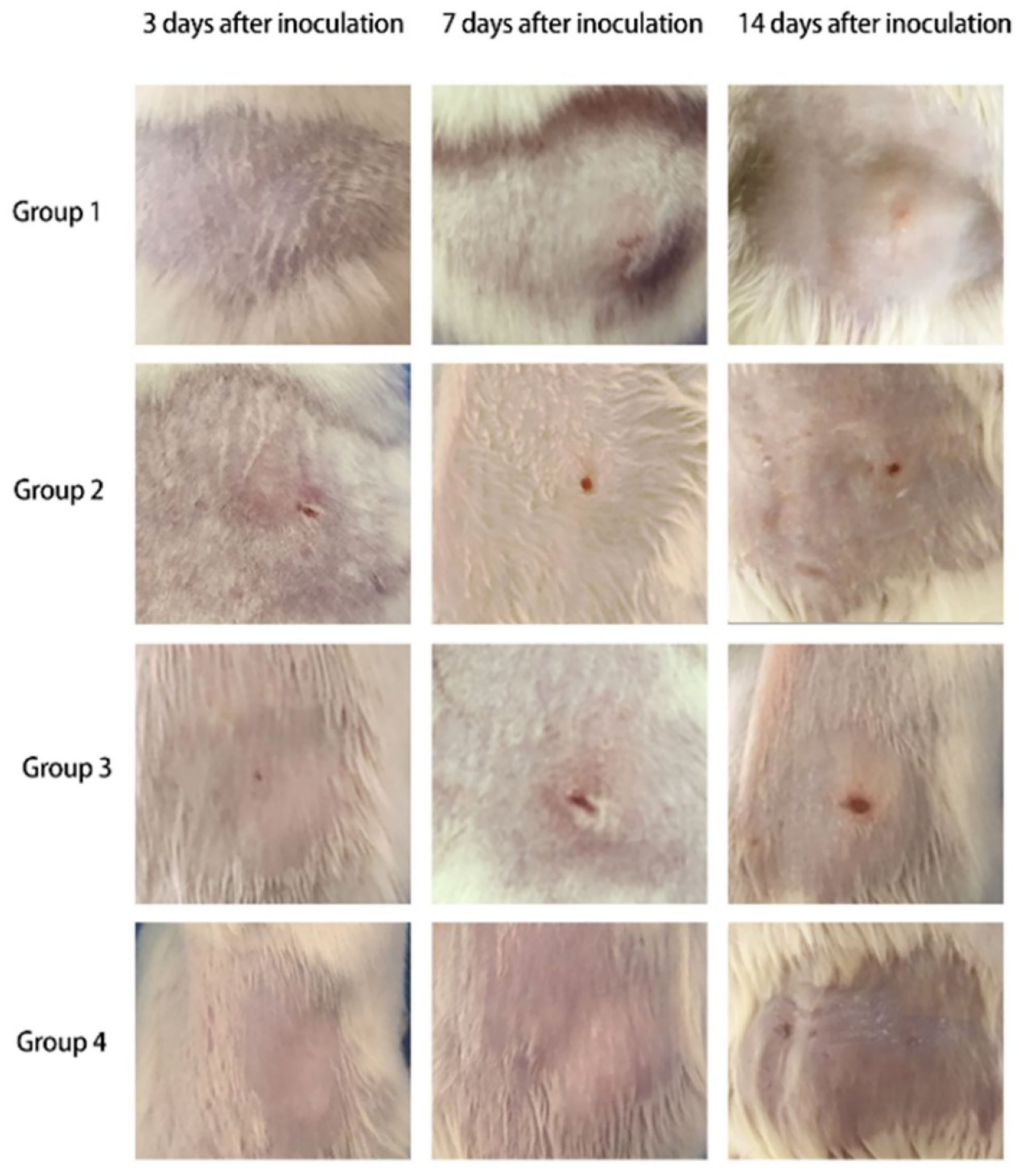
Skin changes of mice in each group (Group 1: CTX 80 mg/kg·d, Group 2: CTX 90 mg/kg·d, Group 3: CTX 100 mg/kg·d, Group 4: CTX 100 mg/kg·d as the control group).

Skin changes and cutaneous ulcers were successfully induced in mice of each group (Group 1: CTX 80 mg/kg·d, Group 2: CTX 90 mg/kg·d, Group 3: CTX 100 mg/kg·d, Group 4: CTX 100 mg/kg·d as the control group). The development of mouse skin lesions in each group can be observed in Figure 1.

- 3 days after inoculation

(a) Group 1: The mental state showed no obvious changes compared with before, and soybean papules could be observed at the skin injection site, with no ulcers.
(b) Group 2: The mental state showed no obvious changes compared with before, and soybean papules could be observed at the skin injection site with a small ulcer on top.
(c) Group 3: The mental state showed no obvious changes compared with before, and a needle tip size ulcer could be observed at the skin injection site.
(d) Group 4: The mental state showed no obvious changes compared with before, and skin color papules could be observed at the skin injection site.

- 7 days after inoculation

(a) Group 1: The mental state of the mice was slightly worse than before, and nodules could be observed at the skin injection site with an ulcer on top.
(b) Group 2: The mental state of the mice was slightly worse than before, and the original ulcer at the skin injection site was larger than before, the base was flushed, and there was a small amount of exudation.
(c) Group 3: The mental state of the mice was slightly worse than before, and the original ulcer at the skin injection site was larger than before, with skin hyperplasia around the ulcer.
(d) Group 4: The mental state of the mice showed no obvious changes compared with before, and the original papule was smaller than before.

- 14 days after inoculation.

(a) Group 1: The mental state of the mice was slightly worse than before, and nodules could be observed at the skin injection site with a light red ulcer on top.
(b) Group 2: The mental state of the mice was worse than before, and the original ulcer was further enlarged, the base was flushed, and exudation increased.
(c) Group 3: The mental state of the mice was worse than before, the original ulcer at the skin injection site was obviously larger than before, exudation increased, and the base was flushed.
(d) Group 4: The mental state showed no obvious changes compared with before, and the original papule was smaller than before.

### Fungal Culture

**Figure 2:**
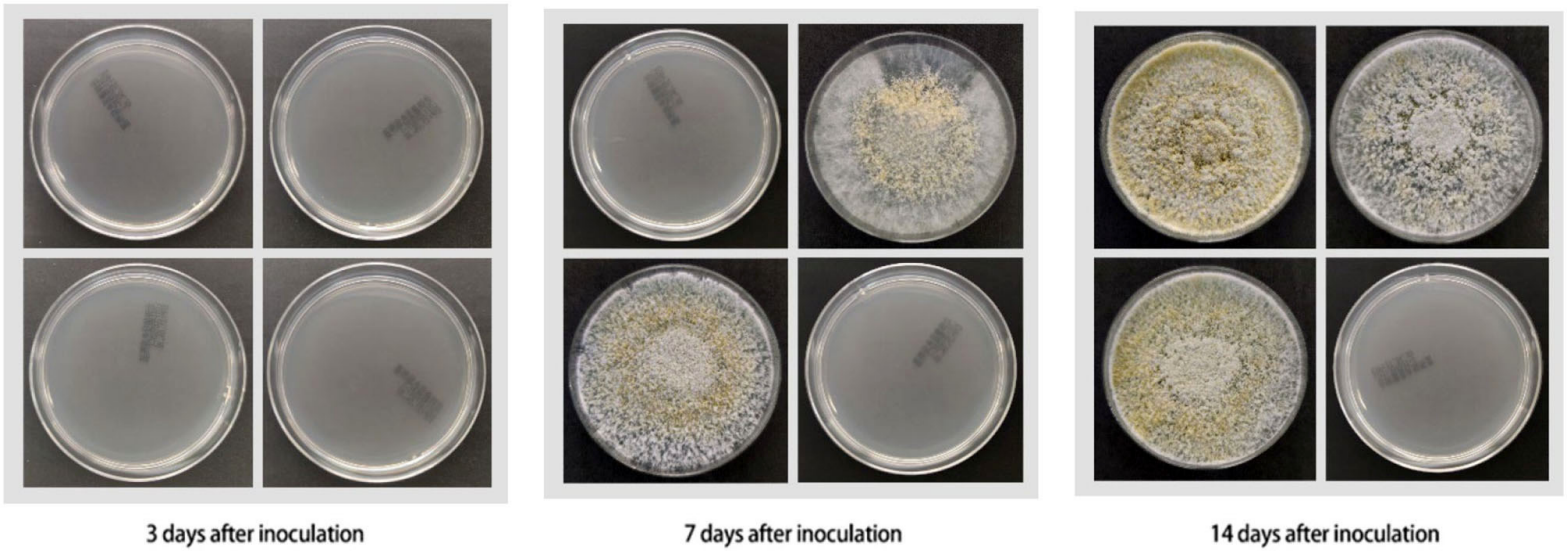
Results of fungal culture of mouse skin tissues (all cultured for 1 week) (from left to right and top to bottom: Group 1, Group 2, Group 3, control group).

The fungal culture results of mouse skin tissue are shown in Table 1 and Figure 2. Mice in all Groups 3 days after inoculation were negative. Mice in all experimental Groups 14 days after inoculation were positive. Mice in Groups 2 and 3 were positive 7 days after inoculation. Mice in the control group were all negative throughout the experiment.

**Table 1:** Results of tissue fungal cultures of mice inoculated with *Trichoderma longibrachiatum* (PKUT180420015) in each group.

**Groups (cyclophosphamide)**	**Culturing results (3 days)**	**Culturing results (7 days)**	**Culturing results (14 days)**
Group 1 (80 mg/kg•d)	Negative	Negative	Positive
Group 2 (90 mg/kg•d)	Negative	Positive	Positive
Group 3 (100 mg/kg•d)	Negative	Positive	Positive
Control group	Negative	Negative	Negative

### Pathological and Immunohistochemical Manifestations

**Figure 3:**
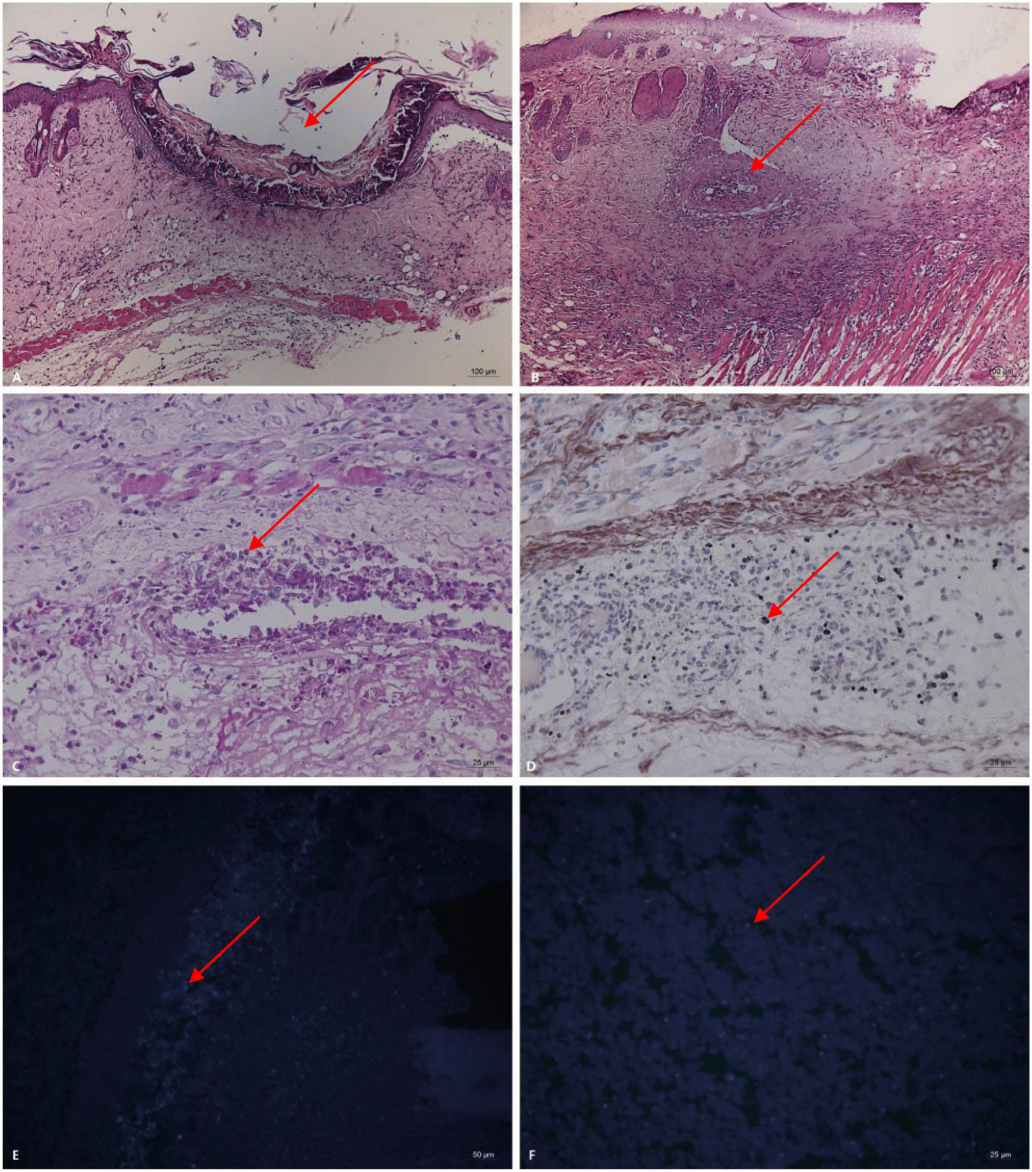
Pathogenicity in the mouse models of *Trichoderma longibrachiatum* (PKUT180420015) from pulmonary spindle cell carcinoma (**A–F**: 14 days after inoculation in Group 3). **(A)** Ulcer formation (arrow) and inflammatory cells increase and accumulate (HE). **(B)** Vasculitis (arrow) around the ulcer (HE). **(C)** Fungal spores (arrow) in and around the blood vessels, and the blood vessel wall is red and necrotic (periodic acid-Schiff staining, original magnification ×400). **(D)** Fungal spores (arrow) in the vascular lumen and vascular wall (periodic acid-silver methenamine). Panel **(E,F)** shows fungal spores (arrow) at the inoculation site and necrotic tissue (Calcofluor staining).

**Figure 4:**
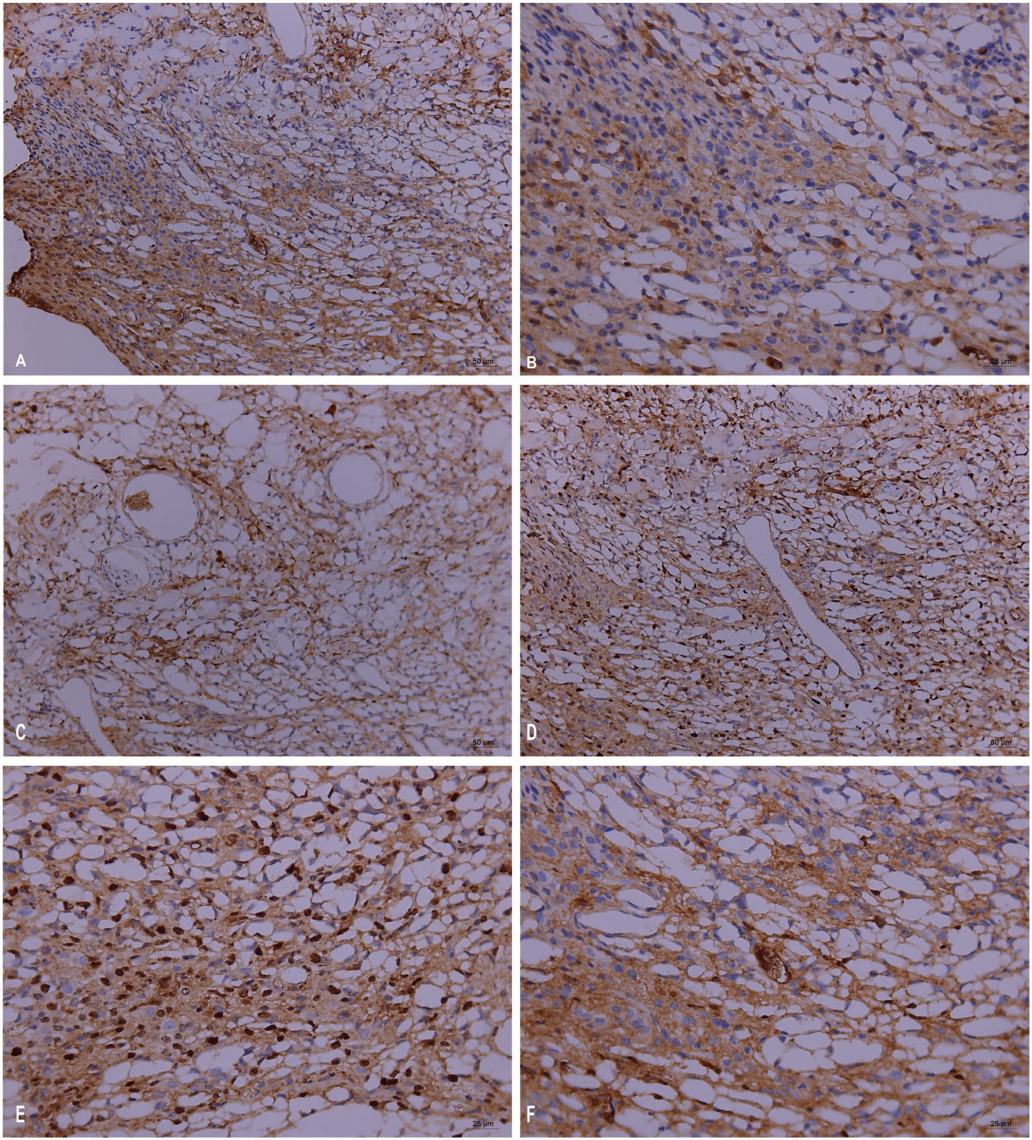
Immunopathology in the mouse models of *Trichoderma longibrachiatum* (PKUT180420015) from pulmonary spindle cell carcinoma: atypical dysplasia with positive staining of CD3ε **(A)**, CD56 **(B)**, GZMB **(C)**, Ki67 **(D,E)**, and PRF **(F)** by immunohistochemistry.

Pathogenicity in the mouse models of *Trichoderma longibrachiatum* infection included ulcer formation, inflammatory cell increases and accumulation, vasculitis, and fungal elements, which were seen in inflamed and necrotic tissue, around the blood vessels, and around the blood vessel wall (periodic acid-Schiff staining, periodic acid-silver methenamine and Calcofluor staining) (Figures 3A–F). In immunohistological staining, atypical dysplasia cells showed expression of CD3ε, CD56, GZMB, Ki67, and PRF (Figures 4A–F).

## Discussion

*Trichoderma* Pers. is a fungus that exists in nature worldwide. Some species of *Trichoderma* spp. can inhibit pathogenic fungi; thus, it is widely used in agricultural production as a biological control agent (7). Since the first case of *Trichoderma* infection was reported in 1970 (8), trichodermasis has now included pulmonary infection, rhino-orbital-cerebral mycosis, oral infection, otitis externa, sinusitis, brain abscess, stomatitis, peritonitis, endocarditis, skin and skin structural infection, keratitis, and septic shock. However, *Trichoderma* infection-associated cancer has not been encountered. We recently reported the isolation of *Trichoderma longibrachiatum* (PKUT180420015) from the tissue of a patient with pulmonary spindle cell carcinoma that was confirmed as a primary infection by laser capture microdissection and polymerase chain reaction (5).

According to the European Organization for Research and Treatment of Cancer and the National Institute of Allergy and Infectious Diseases consensus definitions, our case fulfills the criteria for a “Proven” invasive fungal infection (deep fungal infection) by *T. longibrachiatum*: histopathologic examination showing hyphae from a biopsy specimen with microscopic evidence of associated tissue damage; and growth of *T. longibrachiatum* from a sample obtained by a sterile procedure from a normally sterile and clinically or radiologically abnormal site consistent with infection (microbiological criteria). Periodic acid-Schiff staining, periodic acid-silver methenamine staining, and Calcofluor staining showed fungal spores in the vascular lumen, vascular walls and around the blood vessels. In addition, deep fungal infection caused by *T. longibrachiatum* was later confirmed by laser capture microdissection and polymerase chain reaction (5).

Immunohistochemistry showed positive markers of Ki67, CD3, CD56, GZMB, and PRF, which suggested NK-cell or T-cell infiltration. CD3 is mainly involved in the signal transduction of T cells and consists of four polypeptides, ζ, γ, ε, and δ; thus, CD3ε is mainly used to label T lymphocytes (9). CD56 plays an important role in embryonic development and the interconnection of nerve cells and is used to identify NK cells, but some cells with T-cell markers CD3 and CD4 also express CD56 (10). Perforin is a pore-forming protein that promotes the entry of granulase and other cytotoxic serine proteases into target cells. Perforin is mainly expressed in cytotoxic T lymphocytes and NK cells (11). Granzyme B belongs to the family of serine proteases expressed by cytotoxic T lymphocytes and NK cells and is a key component of immune responses to pathogens and transformed cells (12). Ki67 can be used to detect proliferating cells in G1 phase, S phase, G2 phase and mitotic phase but not in G0 resting phase and is mainly used for evaluating tumor cell proliferation (13). Therefore, Trichoderma infection leads to the aggregation of T cells and NK cells, which are involved in the inflammatory process of *Trichoderma* infection.

NK/T cells are important for host resistance to microbial infection. Recent studies have shown that these cells can aggregate or proliferate in *Mucor irregularis* and *Rhizopus arrhizus* infections that resolve after antifungal therapy alone (14, 15). Further studies showed that *Mucor irregularis* and *Rhizopus arrhizus* could induce NK/T-cell infiltration (CD3+, CD8+, CD56+, TIA1+, GZMB+, PRF+), proliferation (Ki67+), and angioinvasion in mice (6, 14). Recently, Schmidt et al. demonstrated that both interleukin-2-prestimulated and unstimulated human NK cells could damage Rhizopus oryzae hyphae, which corresponds with mucoramycosis (16, 17). The case presented here showed that the *Trichoderma longibrachiatum* strain (PKUT180420015) could also induce NK/T-cell infiltration.

With the increasing number of immunodeficient patients in recent years, the spectrum of opportunistic pathogenic fungi has expanded, and *Trichoderma* spp., which were once considered non-pathogenic, are becoming increasingly worthy of clinical attention. *Trichoderma* infections usually occur in immunocompromised patients. Among immunodeficient patients, Trichoderma strains can cause various diseases ranging from local infections to fatal disseminated diseases. Our study shows that PKUT180420015 isolated from lung tumors can induce mice to express CD3, CD56, perforin, Granzyme B and Ki67 markers. Further investigation of the mechanism of *Trichoderma longarm-*related proliferation is needed.

*Trichoderma* spp. are resistant to amphotericin B, itraconazole, ketoconazole and other traditional antifungal drugs (18). Therefore, it is necessary to actively carry out drug sensitivity tests for Trichoderma infection in clinical practice.

In summary, our studies have shown that the *Trichoderma longibrachiatum* strain (PKUT180420015) isolated from a biopsy specimen of a patient with pulmonary spindle cell carcinoma could induce atypical hyperplasia, with the expression of Ki67, CD3, CD56, GZMB, and PRF in mice, indicating that the fungus may be involved in inducing tumorigenesis.

## Data Availability Statement

The original contributions presented in the study are included in the article/supplementary material, further inquiries can be directed to the corresponding author.

## Ethics Statement

The animal study was reviewed and approved by Peking University Third Hospital IRB; approval #00006761-2015025.

## Author Contributions

GZ performed the experiment and wrote the manuscript. DL conceptualized the experiment, identified the fungus, performed pathological diagnosis, and revised the manuscript. All authors contributed to the manuscript revision and read and approved the submitted version.

## Funding

We gratefully acknowledge the financial support by the National Natural Science Foundation of China (31770013, 81571967, and 32070019).

## Conflict of Interest

The authors declare that the research was conducted in the absence of any commercial or financial relationships that could be construed as a potential conflict of interest.

## Publisher's Note

All claims expressed in this article are solely those of the authors and do not necessarily represent those of their affiliated organizations, or those of the publisher, the editors and the reviewers. Any product that may be evaluated in this article, or claim that may be made by its manufacturer, is not guaranteed or endorsed by the publisher.
